# DESIGN AND USE OF ROBOTS TO ASSIST OLDER ADULTS WITH HEALTHCARE TASKS

**DOI:** 10.1093/geroni/igz038.126

**Published:** 2019-11-08

**Authors:** Wendy Rogers, Megan Bayles

**Affiliations:** University of Illinois Urbana-Champaign, Champaign, Illinois, United States

## Abstract

There is much potential for robots to support the needs of older adults, in general, and particularly in healthcare. Older adults are quite open to the idea of interacting with robots, although they have preferences for the nature of the task they want the robot to do as well as what they want the robot to look like. These preferences should be considered in the process of design and deployment. Older adults should be involved throughout the design process from formative to summative evaluation and even beyond to the integration of the robot into their everyday activities. The extant research provides guidance regarding older adults’ capabilities and limitations that might influence their ability to interact with a robot. Our goal in this presentation will be to focus on robots being designed to support older adults with healthcare tasks in the context of enhanced, instrumental, and basic activities of daily living.

